# Improving the Continuous Microcellular Extrusion Foaming Ability with Supercritical CO_2_ of Thermoplastic Polyether Ester Elastomer through In-Situ Fibrillation of Polytetrafluoroethylene

**DOI:** 10.3390/polym11121983

**Published:** 2019-12-02

**Authors:** Rui Jiang, Tao Liu, Zhimei Xu, Chul B. Park, Ling Zhao

**Affiliations:** 1 State Key Laboratory of Chemical Engineering, East China University of Science and Technology, Shanghai 200237, China; 030130876@mail.ecust.edu.cn (R.J.); liutao@ecust.edu.cn (T.L.); zhmxu@ecust.edu.cn (Z.X.); 2 Microcellular Plastics Manufacturing Laboratory, Department of Mechanical and Industrial Engineering, University of Toronto, Toronto, ON M5S 3G8, Canada; park@mie.utoronto.ca; 3 College of Chemistry and Chemical Engineering, Xinjiang University, Urumqi 830046, China

**Keywords:** supercritical carbon dioxide, extrusion foaming, in-situ fibrillation, TPEE

## Abstract

In-situ fibrillated polytetrafluoroethylene (PTFE) enhanced nanocomposites were successfully prepared by mixing thermoplastic polyether ester elastomer (TPEE) and PTFE using a twin-screw extruder. Well-dispersed, long aspect ratio PTFE nanofibrils with a diameter of less than 200 nm were generated and interwoven into networks. Differential scanning calorimetry and in-situ polarized optical microscopy showed that the PTFE nanofibrils can greatly accelerate and promote crystallization of the TPEE matrix and the crystallization temperature can be increased by 6 °C. Both shearing and elongational rheometry results confirmed that the introduction of PTFE nanofibrils can significantly improve the rheological properties. The remarkable changes in the strain-hardening effect and the melt viscoelastic response, as well as the promoted crystallization, led to substantially improved foaming behavior in the continuous extrusion process using supercritical CO_2_ as the blowing agent. The existing PTFE nanofibrils dramatically decreased the cell diameter and increased cell density, together with a higher expansion ratio and more uniform cell structure. The sample with 5% PTFE fibrils showed the best foaming ability, with an average diameter of 10.4–14.7 μm, an expansion ratio of 9.5–12.3 and a cell density of 6.6 × 10^7^–8.6 × 10^7^ cells/cm^3^.

## 1. Introduction

Thermoplastic elastomers (TPEs) are copolymers composed of crystalline rigid segments and amorphous soft segments. They have experienced a market boom during recent decades and the rising trend will continue due to their remarkable thermal, chemical and mechanical properties [1]. TPEs can be easily manufactured by extrusion, injection molding, thermoforming and spinning [2] and can be considered a bridge between thermoplastics and chemically crosslinked elastomers [3].

Thermoplastic polyether ester elastomer (TPEE) is a novel class of TPE, which has proved to be a potential material to replace rubbers [4]. In general, the rigid segments in TPEE are aromatic semi-crystalline polyesters that have a high melting temperature to ensure physical crosslinking between the repeating soft segments. The soft segments are polyethers with a rather low glass transition temperature, such as poly(tetramethylene glycol) (PTMG) [5], poly(tetramethylene oxide) (PTMO) [6] and poly(ethylene glycol) (PEG) [7], to ensure flexibility, ductility and elasticity. TPEE has many excellent properties including thermal stability, good elasticity, chemical and oil resistance and, especially, rebound resilience at low-temperatures [3,5]. Also, TPEE has higher tear and impact strengths over a broad range of service temperatures when compared with other traditional TPEs [8]. Because of the extraordinary performance of TPEE, it has attracted increasing interest from many fields such as the sports, automotive and military industries.

Although TPEE is an attractive engineering material, its application has to overcome the exorbitant price. To overcome this shortcoming, many attempts have been made to further improve the mechanical properties or to lower the weight (i.e., material consumption). Blending the matrix with micro-sized particles is an efficient method to improve the thermal or mechanical properties of TPEE [9,10,11,12,13]. Paszkiewicz et al. [12] added carbon nanotubes (CNTs) and graphene nanoplatelets (GNPs) to poly(trimethylene terephthalate) (PTT)-based TPEE to improve the electrical conductivity. They found that CNTs had greater potential to improve electrical conductivity when compared to GNPs due to the higher purity and higher aspect ratio. Qiu et al. [13] controlled the structure of mixed filler particles in TPEE to enhance the mechanical properties. The results showed that a closely packed particle structure can significantly improve the yield strength by about 40% with a decrease of about 20% in the Young’s modulus, which is preferred in TPEE used as an elastomer.

Foaming is a very effective way to lower the weight and expand the application of polymers. In recent decades, foamed materials have exhibited a balance between price and superior properties, such as better heat insulation, light weight, acoustic insulation and impact resistance [14,15,16]. General processes to prepare polymer foams include batch foaming [17], steam chest molding foaming [18], extrusion foaming [19] and injection molding foaming [20]. Compared with other approaches, extrusion foam processing can prepare polymer foams in a continuous way, which is economically friendly and easy to scale-up. However, it may meet many more challenges when considering the melt properties of polymers. In particular, extrusion foaming requires high melt strength, typically from a branched molecular structure and a strain-hardening response in extensional flow to have a broad processing window [21,22]. However, raw TPEE cannot be used for extrusion foaming due to its low melt strength and low viscoelasticity. To overcome the low melt strength of linear polymers, chemical modification or electron beam crosslinking can be used as a common strategy to improve foaming ability [21,23,24]. Unfortunately, these efficient processes require a radioactive source or reactor which may increase the cost of the product. Moreover, the polymer may be over-crosslinked and turn into gel, which is non-recyclable and cannot be used in the extrusion foaming process [25]. Dispersed micro/nanoparticles such as modified layered nanoclay and carbon particles can act as nucleating agents to increase the cell density in extrusion foaming [22,26] but the blends show little strain hardening response in extensional flow, which is important to stabilize the cell structure. Moreover, uniform dispersion of nanoparticles is extremely difficult and challenging, although particle surface modification that requires more processing can improve the dispersion.

Recently, it was observed that introducing micro-fibrils into a polymer matrix can be a new method to enhance the foaming ability [27,28,29,30,31]. Polypropylene (PP) reinforced with fibrillated Polyethylene terephthalate (PET) and Polyethylene (PE) reinforced with fibrillated PP were successfully prepared by Rizvi et al. using a fiber-spun system [27,28]. In these works, the well dispersed polymer fibrils could significantly enhance the extensional rheological properties of the matrix and hinder cell coalescence, resulting in a higher cell density and a higher expansion ratio. However, the materials in this process went through three steps of heating, that is, melt blending, spinning and matrix re-melting. Potentially, degradation can occur in these processes, especially for polyesters. A one-step process to prepare fibril reinforced polymer composites has been demonstrated in recent years using Polytetrafluoroethylene (PTFE) fibrils [32,33]. PTFE is a highly crystalline polymer with an extremely low coefficient of friction and low density of chain entanglements, thus in high shearing flow it tends to be stretched into large-aspect-ratio fibril structures and even forms network structures in the matrix polymer [34]. Due to the well-dispersed PTFE fibrils, the melt strength can be significantly improved. Rizvi et al. and Zhao et al. used PTFE fibrils in extrusion foaming and foam injection-molding of PP, respectively. They found that in-situ fibrillated PTFE could increase the solubility of CO_2_ in the blend and provide strain-induced hardening behavior in the foaming process, which can increase cell density and stabilize the existing cells. Huang et al. found that fibrillated PTFE altered the viscoelastic properties and improved the foaming property of TPU/PTFE composites in foam injection-molding [29].

In this research, an in-situ fibrillation process was used to prepare TPEE/PTFE blend to improve the foaming ability of neat TPEE in extrusion processing. Highly expanded TPEE foams were successfully obtained with the added PTFE fibrils. First, a twin-screw extruder was used to prepare the PTFE nanofibril reinforced TPEE nanocomposite. The dispersion of PTFE and the structure of the fibrils were observed by a scanning electron microscope (SEM). Shearing and elongational rheological tests were performed to study the rheological behavior of the TPEE melt. Differential scanning calorimetry (DSC) and polarized light microscopy (POM) were used to investigate the crystallization of the blends. TPEE foams were prepared using the TPEE/PTFE fibril nanocomposites by extrusion foaming. With the addition of PTFE fibrils, the TPEE/PTFE blends showed excellent foaming ability with a high expansion ratio and a finer cell structure.

## 2. Experimental Section

### 2.1. Materials

A commercially available linear TPEE segment copolymer supplied by Sichuan Sunplas Inc. (Chengdu, China), H6055, was used as the matrix without any further treatment. The melt flow rate (MFR) was 13 g/10 min (with a load of 2.16 kg at 230 °C) and the melting temperature was about 195 °C. 2,2′-bis(2-oxazoline) (2,2′-BOZ) was purchased from Tokyo Chemical Industry (Tokyo, Japan) and used as received. PTFE powder (Metablen™ A-3000) was supplied by Mitsubishi Rayon Co., Ltd., Tokyo, Japan. TPEE pellets and PTFE powder were dried at 110 °C in a vacuum drying box overnight and were then well mixed with a given mass ratio. To increase the viscosity of commercial linear TPEE, 0.5 wt % 2,2′-BOZ was added to all the nanocomposites, as mentioned in previous studies [35,36]. Table 1 shows the compositions of the nanocomposites. CO_2_ and N_2_ were purchased from Air Product Co., Shanghai, China and used as a physical blowing agent and a protection gas, respectively.

### 2.2. Nanocomposite Preparation and Characterization

A co-rotating twin-screw extruder (SHJ-20, Nanjing Giant Co., Nanjing, China) with a screw diameter of 20 mm and an length-diameter (L/D) ratio of 30 was used to compound the TPEE/PTFE composites with four different weight fractions, as listed in Table 1. With a PTFE content higher than 5%, the melt elasticity of the extrudate was really high, such that it could not be extruded continuously. Therefore, in this study, the PTFE contents used were 1%, 3% and 5%. The extruder barrel temperature profile from the hopper zone to the die was 200, 215, 215, 215, 215 and 210 °C. The feed rate was set at 3 kg/h and the screw speed was set at 200 rpm to achieve uniform dispersion and in-situ fibrillation of PTFE powder in the TPEE matrix. The products were cooled and cut into pellets. The pellets were dried completely before characterization and extrusion foaming.

### 2.3. Extrusion Foaming of TPEE/PTFE Fibril Nanocomposites

**Figure 1:**
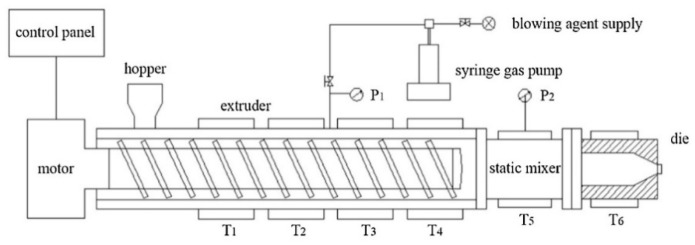
Schematic representation of the foaming system.

A foam extrusion system comprised of a single-screw extruder (Hangzhou Jinghai Plastics Technology Co., Hangzhou, China) and a CO_2_ gas injection pump (SFT-10 of Septech Ltd., Shanghai, China) was used for extrusion foaming, as shown in Figure 1. The diameter of the screw was 50 mm and the aspect ratio (length/diameter) was 45. A cylindrical die with a diameter of 1.5 mm and a channel length of 6 mm was fitted at the extruder outlet. The temperature along the extruder from *T*_1_ to *T*_4_ was set at 160, 190, 200 and 200 °C. The temperature of the static mixer (*T*_5_) and die (*T*_6_) was set ranging from 175 to 190 °C. The screw speed was 35 rpm and 1 wt % CO_2_ was injected between the second and third extruder barrel zones after the compression zone, where the pellets would most probably be totally melted.

### 2.4. Morphological Characterization of the TPEE/PTFE Nanocomposites

An environmental scanning electron microscope (SEM, FEI QUANTA FEG 250, Hillsboro, OR, USA) was used to observe the morphology of the dispersed PTFE fibrils in the TPEE matrix. The TPEE/PTFE nanocomposites with different PTFE contents were hot-pressed into sheets and cryogenically fractured in liquid nitrogen. The fractured samples were exposed to trifluoroacetic acid vapor at 60 °C for 30 min to selectively remove part of the TPEE and expose the PTFE fibrils in the TPEE matrix.

### 2.5. Rheological Tests

The shear rheological properties of all the extruded products were measured using a rheometer (HAAKE MARS III, Waltham, MA, USA) under a nitrogen atmosphere. A 25 mm parallel-plate configuration with a 1.8 mm gap was selected for the measurements. A strain of 3% within the linear viscoelastic range was used for small amplitude oscillatory shear tests. The angular frequency scanned was from 100 rad/s to 0.1 rad/s at 230 °C.

The elongational viscosity of the extruded products was also measured using the same rheometer (HAAKE MARS III, Waltham, MA, USA) equipped with an extensional viscosity fixture. The samples were hot-pressed and cut into sheets (20 mm long, 10 mm wide and 0.8 mm thick). The measurements took place at 205 °C under a nitrogen atmosphere with strain rates of 0.01 s^−1^, 0.05 s^−1^, 0.1 s^−1^, 1 s^−1^ and 0.5 s^−1^.

Before the shear and elongational measurements, the samples were preheated to the designated temperature and held for 5 min to ensure the samples were completely molten.

### 2.6. Thermal Analysis

The melting point and thermal stability of PTFE powder were tested with a differential scanning calorimeter (DSC, NETZSCH HP204, Selb, Germany) and a thermogravimetric analyzer (TGA, TA Q50, New Castle, DE, USA), respectively. For the DSC test, the sample was heated from 35 to 400 °C at a rate of 10 °C /min under a nitrogen atmosphere. For the TGA test, the sample was heated from 35 to 600 °C at a rate of 20 °C/min under a nitrogen atmosphere.

The non-isothermal crystallization kinetics of the composites was studied by the same differential scanning calorimeter (DSC, NETZSCH HP204, Selb, Germany). The crystallization process involved heating the sample from room temperature to 230 °C at a rate of 10 °C/min and holding for 3 min to erase the thermal history. The samples were then cooled to 100 °C at a rate of 5 °C/min to record their crystallization behavior.

### 2.7. Polarized Optical Microscopy (POM) Measurements

A polarized optical microscope (POM, Olympus BX51, Tokyo, Japan) equipped with a hot stage was used to observe the morphology of the TPEE/PTFE nanocomposites. The samples were placed in a two-piece micro cover glass and heated to be completely molten at 210 °C. Then, the stage was rapidly cooled to 180 °C to observe the TPEE crystallization process.

### 2.8. Foam Characterization

A scanning electron microscope (SEM) (JSM-6360LV, Tokyo, Japan) was adopted to show the cell structure of foamed samples. The samples were prepared by breaking in liquid nitrogen and coated with platinum before observation. The average cell diameter was calculated by Image-Pro Plus software based on the SEM figures. Cell density—*N*_0_ (cells/cm^3^)—was determined by Equation (1) [37].

(1) $$N_{0}={\left(\frac{n}{A}\right)}^{3/2}\times R_{v}$$

where *n* represents the number of pores in the SEM figure, *A* (cm^2^) represents the area of the figure. *R*_v_ represents the expansion ratio, which is defined as the ratio of the sample density before and after foaming and was calculated by Equation (2).

(2) $$R_{v}={\rho_{0}/\rho }_{f}$$

where *ρ*_0_ and *ρ_f_* represent the densities of the samples before and after foaming. A displacement method was employed to measure the density according to ASTM D792-13.

## 3. Results and Discussion

### 3.1. Morphology of TPEE/PTFE Nanocomposites

**Figure 2:**
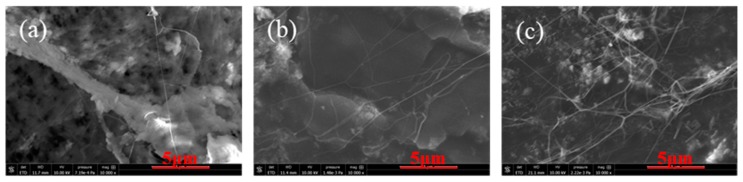
Scanning electron microscope (SEM) images of the morphology of PTFE fibrils in TPEE matrix: (**a**) TPEE with 1% PTFE (PTFE1), (**b**) TPEE with 3% PTFE (PTFE3) and (**c**) TPEE with 5% PTFE (PTFE5).

Figure 2 shows the fractured surfaces of TPEE with different PTFE contents. To expose the PTFE phase, all the fractured surfaces were etched by trifluoroacetic acid vapor to partly remove the TPEE matrix. Obviously, the uniformly distributed PTFE phase shows a fibrillated structure in the TPEE matrix and the diameter of PTFE fibrils in this work is less than 200 nm, thus the aspect ratio can be extremely high. Many fibrils are entangled together to generate a reticular structure, which is believed to enhance the melt strength and prevent cells from rupture in the foaming process. Zhao’s work shows that highly fibrillated PTFE could improve the foaming ability of PP in the injection-molding foaming process [30,38].

### 3.2. Thermal Properties of PTFE Fibrils

**Figure 3:**
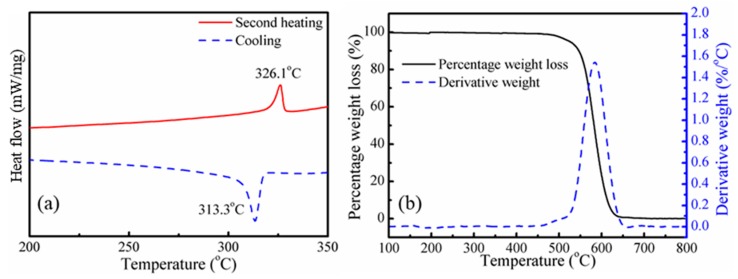
Thermal properties of PTFE powder: (**a**) cooling and second heating differential scanning calorimetry (DSC) curve, (**b**) thermogravimetric analysis (TGA) and derivative thermal gravimetry (DTG) curves.

The thermal properties of PTFE powder were studied by DSC and TGA, as shown in Figure 3. According to Figure 3a, the melting temperature and crystallization temperature of PTFE powder are 326.1 and 313.3 °C, respectively, which are much higher than the processing temperature in this work. The thermal degradation behavior of PTFE powder was studied by thermogravimetric analysis (TGA) and derivative thermal gravimetry (DTG). The TGA and DTG curves are shown in Figure 3b. The temperature at which 5% (*T*_5%_) degradation occurs and the maximum mass loss temperature (*T*_max_) are 525 and 584 °C, respectively. Thus, during the blending process, no melting or degradation of PTFE would occur. It can be confirmed that the PTFE fibrils were in-situ fabricated from PTFE powder directly in the extrusion blending process. The mechanism of in-situ fibrillated PTFE results from the high crystallinity and low lattice energy when undergoing a strong shearing flow field; the PTFE particles could be stretched into microfiber or even nanofibrils during the processing [33,34].

### 3.3. Thermal Behavior of the TPEE/PTFE Nanocomposites

**Figure 4:**
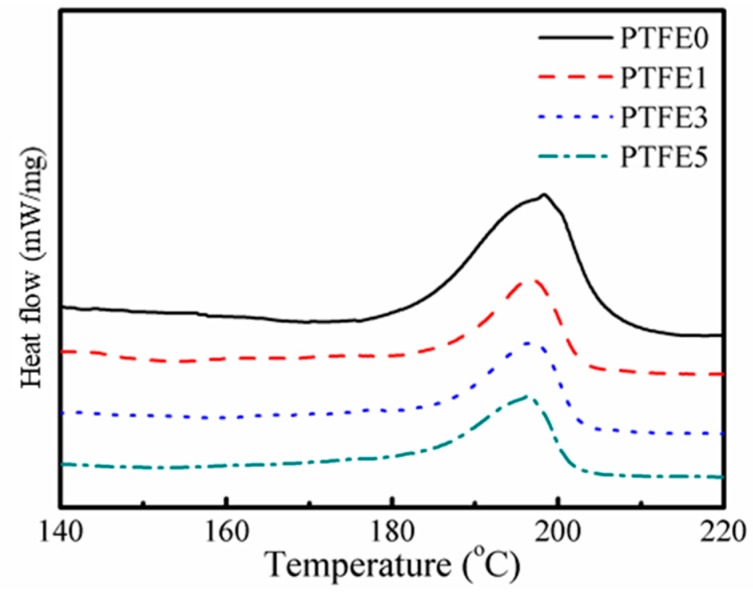
Second heating curves of TPEE/PTFE nanocomposites.

The thermal behavior of the TPEE/PTFE nanocomposites was studied by DSC and both melting and crystallization processes were taken into consideration. Figure 4 shows the second heating curves of TPEE/PTFE nanocomposites with a heating rate of 10 °C/min. After eliminating the thermal history of the samples, all the samples have a similar melting point at around 197 °C. However, the samples with PTFE nanofibrils show a slightly lower melting enthalpy, indicating lower crystallinity due to the hindering effect of the nanofibrils.

**Figure 5:**
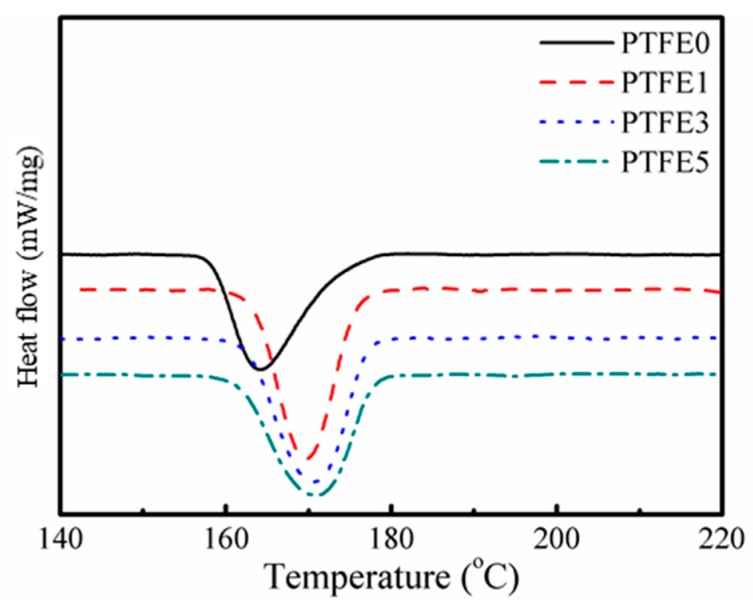
Cooling curves of TPEE/PTFE nanocomposites.

Interestingly, as shown in Figure 5, compared with TPEE without PTFE nanofibrils, the crystallization temperature of TPEE with PTFE nanofibrils increases by approximately 6 °C, from 164.3 °C for TPEE without PTFE and to 169.6, 170.6 and 170.8 °C for TPEE with 1%, 3% and 5% PTFE, respectively, demonstrating that the fibrillated PTFE can act as a nucleation agent to promote the crystallization of TPEE with a high degree of close packing. The work of PP/PTFE system also showed that introducing PTFE fibrils can greatly increase the crystallization temperature by at least 6 °C [30]. To qualitatively investigate promotion of PTFE fibrils in the TPEE crystallization kinetics, a modified Avrami analysis was employed, as described in earlier works [39] and the region calculated ranged from 3% to 50% in relative crystallinity to avoid the influence of epiphytic crystal growth. The crystallization kinetic parameters (*k’* and *n*), together with the enthalpy of crystallization (Δ*H_c_*), the crystallization onset temperature (*T*_c, onset_), the crystallization temperature (*T*_c_), the melting temperature (*T*_m_) and the half-crystallization time (*t*_1/2_, defined as when the relative crystallinity reaches 50%) are listed in Table 2. *r*^2^ is the variance in the statistics.

As illustrated in Table 2, the introduction of PTFE nanofibrils can greatly promote the crystallization of the TPEE matrix. Both the crystallization onset temperature and the crystallization temperature are higher compared to the sample without PTFE nanofibrils. Moreover, the shorter half-crystallization times and the higher Avrami indexes *n* of the nanocomposites indicate that the crystallization process was significantly accelerated. The presence of PTFE fibrils can also hinder the growth of TPEE crystals because of the increased crystal-to-crystal interactions [40,41], resulting in lower crystallinity compared to the sample without PTFE nanofibrils. Thus, sample PTFE5 has the lowest crystallinity, the lowest Avrami index and the longest half-crystallization time of all the TPEE/PTFE nanocomposites.

**Figure 6:**
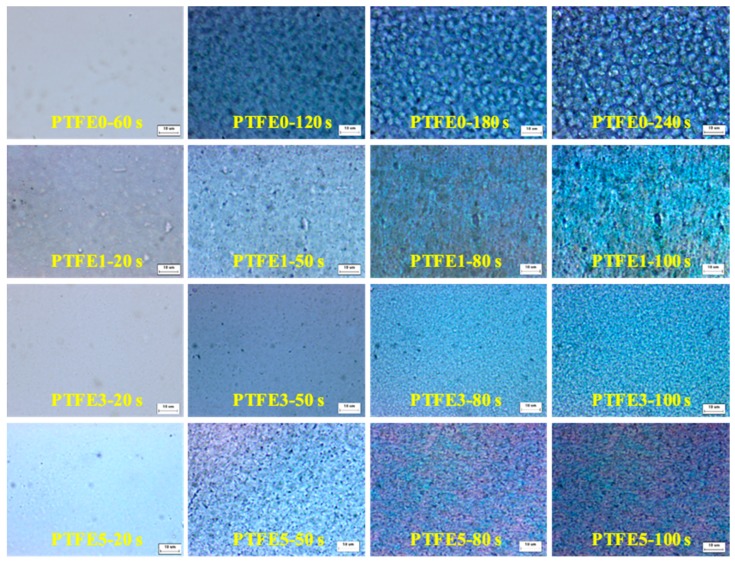
POM images of TPEE/PTFE nanocomposites isothermally crystallized at 180 °C (the scale bar represents 10 μm).

**Figure 7:**
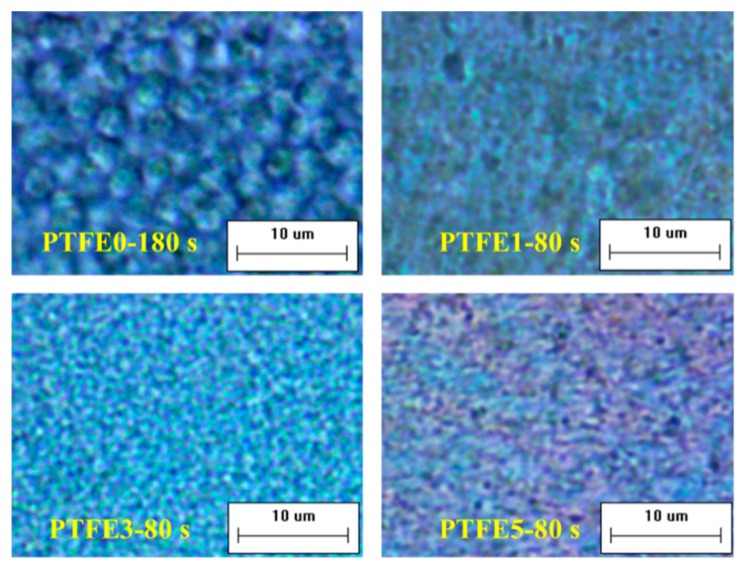
High magnification POM images of TPEE/PTFE nanocomposites. (the scale bar represents 10 μm.).

The difference in evolution of crystal growth was investigated at 180 °C by POM equipped with an in-situ observation hot-stage. As shown in Figure 6, pure TPEE shows stable crystal growth and the crystals are spherical with a diameter around 5 μm. The whole crystallization time is about 240 s when the generated crystals stop growing. It is clear that, when introducing PTFE nanofibrils, the crystallization process is evidently accelerated. The total crystallization time decreases to around 100 s and, in the early period of this process, many more nucleated crystals can be found in the samples with PTFE nanofibrils, resulting in a much higher crystal number density. In the high magnification POM images shown in Figure 7, with a higher PTFE content, especially for the sample PTFE3 and PTFE5, rod-like crystals with a mean size of less than 1 μm can be observed and these rod-like crystals grow together to generate a crystal network. In recent studies, it was found that in-situ generated fibrils can act, not only as a crystal nucleation agent but also as a crystallization template [42,43]. The crystals can grow along and around the fibrils to form a trans-crystalline layer. In this study, the fibrillated PTFE can also act as a template to change the crystal structure of TPEE from spherical to rod-like. All of these changes proved that PTFE nanofibrils could significantly promote the crystallization of the TPEE matrix, which is in conformity with the DSC analysis.

### 3.4. Rheological Analysis

**Figure 8:**
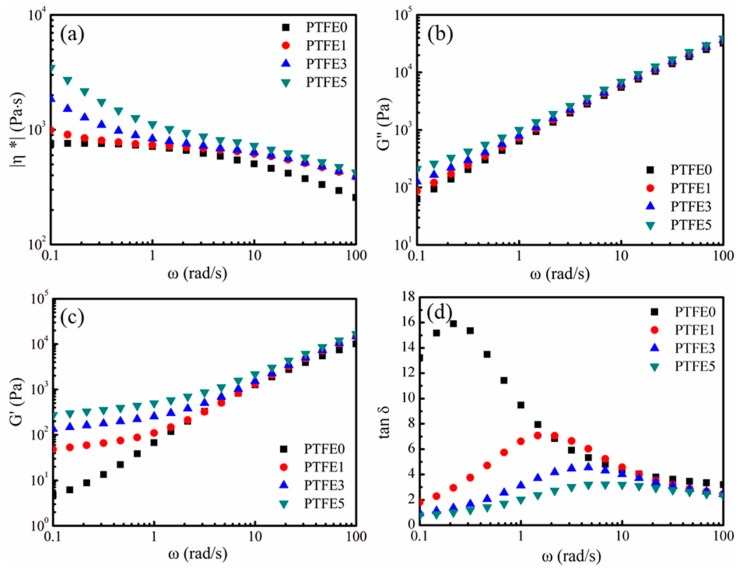
Effect of PTFE nanofibrils on the shearing rheological properties at 230 °C: (**a**) Complex viscosity (|η*|), (**b**) Loss moduli (*G*″), (**c**) Storage moduli (*G*′) and (**d**) Tangential loss angle (tan δ).

In the extrusion foaming process, as well as other melt foaming processes, the rheological properties of the melt play an essential role. To investigate the influence of PTFE nanofibrils on the shearing rheological properties of the nanocomposites, small amplitude oscillatory shear (SAOS) tests were employed in the linear viscoelastic region at 230 °C. The complex viscosity (η^*^) against the shearing frequency (ω) is presented in Figure 8a. Sample PTFE0, which has no PTFE nanofibrils, displays a Newtonian plateau and a lower complex viscosity. Compared with sample PTFE0, the samples which have PTFE nanofibrils show a higher complex viscosity, especially in the low frequency region. The complex viscosity values for samples PTFE0, PTFE1, PTFE3 and PTFE5 at 0.01 rad/s are 752.4, 993.5, 1853.5 and 3467.6 Pa·s, respectively. Moreover, it is observed that the viscosity of the samples with PTFE nanofibrils shows a dramatically rising trend in the low frequency region. The existing PTFE fibrils can be entangled and form a network, which can hinder the mobility of TPEE melts, particularly in the low frequency region.

The loss (*G*″) and storage moduli (*G*′) are plotted against *ω* in Figure 8b,c. Both *G*″ and *G*′ show an increasing trend with increasing PTFE content, especially in the low frequency region, whereas the values of *G*″ and *G*′ for the samples with different PTFE content are almost the same as in the high frequency region. The increasing of *G*″ and *G*′ indicates a longer chain relaxation process, which can enhance the cell nucleation and growth in the foaming process due to the increased local stress [44]. When structural changes occur, *G*′ can be more sensitive than *G*″ [45]. As Figure 8c shows, the values of *G*′ for the samples PTFE1, PTFE3 and PTFE5 deviate from that for sample PTFE0, indicating a strong interaction between the generated PTFE fibrils and the TPEE matrix. Moreover, with a higher PTFE content, such as samples PTFE3 and PTFE5, a platform region can be observed in the low frequency region. Considering that PTFE nanofibrils can interweave into meshes or networks, the strong interactions between the dispersed phase and the continuous phase make the polymer melts show a solid-like or gel-like viscoelastic response. A similar phenomenon can also be found in nanocomposites using clay [45] or montmorillonite [46] as the nanofiller and this gel-like behavior could be ascribed to the generation of a nanofiller network.

The changes of the elastic response for the PTFE nanofibril enhanced TPEE melts are expressed by tan δ, which is calculated by dividing *G*″ by *G*′, and δ is the loss angle. As illustrated in Figure 8d, compared with sample PTFE0, all the samples having PTFE nanofibrils show an obvious decreasing trend in tan δ, especially in the low frequency region, which means that these melts have a higher elastic response. The nanofibrils and the existence of networks can greatly enhance the viscoelasticity of the nanocomposite melts, which can evidently hinder cell coalescence and rupture [31,47].

**Figure 9:**
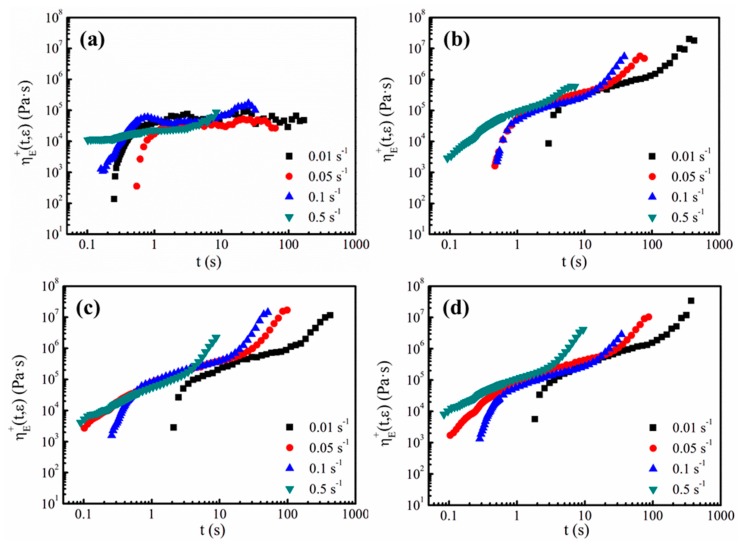
Elongational viscosity of TPEE/PTFE nanocomposites at 205 °C at various elongation rates: (**a**) PTFE0, (**b**) PTFE1, (**c**) PTFE3 and (**d**) PTFE5.

When the cells grow in the foaming process, the cell walls experience biaxial stretching, thus the elongational viscosity of the melts takes an active role in cell growth. In addition to the shear rheological tests, the measurements of the elongational viscosity, $\eta_{E}^{+}(t,\varepsilon )$, at different elongation rates ranging from 0.01 to 0.5 s^−1^ were made for all the samples. Figure 9 displays all the elongational viscosity curves of the TPEE with different PTFE contents. As shown in Figure 9a, due to the lack of branched chains and PTFE nanofibrils, sample PTFE0 shows little strain-hardening behavior [35]. Only for a higher elongation rate such as 0.1 and 0.5 s^−1^, does the elongational viscosity rise with time. In contrast, all the composites with PTFE nanofibrils show pronounced strain-hardening at strain rates between 0.01 and 0.5 s^−1^.

A quantitative analysis was employed to describe the changes in the elongational viscosity in detail. The strain hardening factor, $\chi_{E}$, is defined in Equation (3). $\eta^{+}(t)$ is the transient shearing viscosity and, in this study, $\eta_{E}^{+}(t,\varepsilon )$ is the value when the Hencky strain reaches 2.5 [48].

(3) $$\chi_{E}=\frac{\eta_{E}^{+}(t,\varepsilon )}{3\eta^{+}(t)}$$

**Figure 10:**
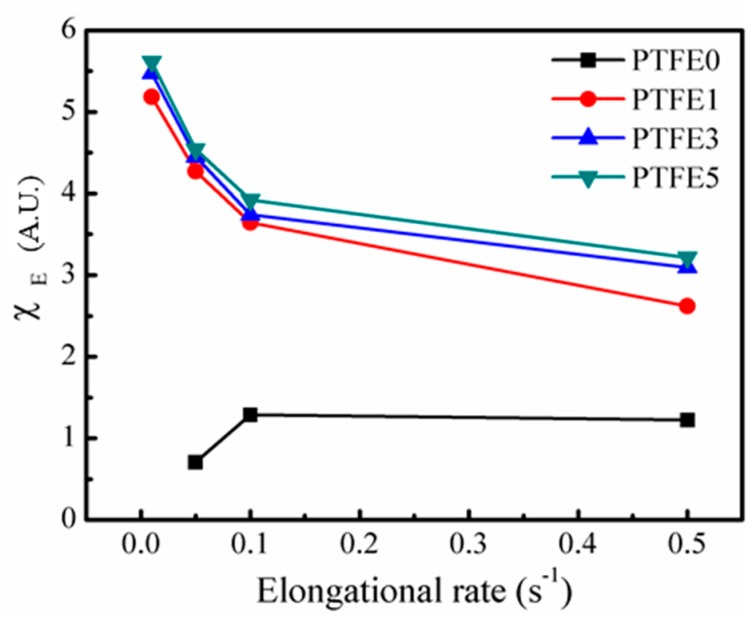
$\chi_{E}$ for TPEE/PTFE nanocomposites with different elongation rates.

Figure 10 shows $\chi_{E}$ with various elongation rates for all the samples. The Hencky strain value cannot reach 2.5 for PTFE0 at a rate of 0.01 s^−1^ and at a rate of 0.05 s^−1^, $\chi_{E}$ is below 1, showing strain-softening behavior. Only at the high elongation rate is $\chi_{E}$ for PTFE0 higher than 1, showing slight strain-hardening behavior. This phenomenon is due to the linear chain structure of TPEE, which was studied in detail in previous work [35], where all the samples with PTFE nanofibrils showed extremely high $\chi_{E}$ values for all the rates employed. As discussed for the shear rheological tests, the entangled network can lead to a long relaxation process [28]. The existing nanofibrils can impede the stretching and flow of TPEE matrix. The significant strain-hardening implies that the introduction of PTFE nanofibrils can change the elongational rheological behavior of TPEE.

### 3.5. Extrusion Foaming of TPEE/PTFE Nanocomposites

**Figure 11:**
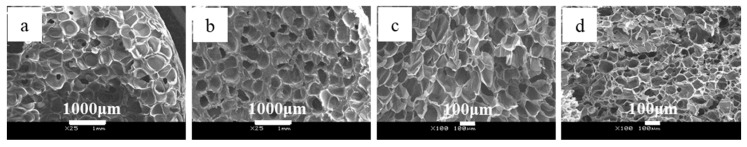
SEM images of extrusion foamed samples obtained at 180 °C (**a**: PTFE0, **b**: PTFE1, **c**: PTFE3, **d**: PTFE5).

Foaming experiments were conducted in a single-screw extruder equipped with a gas injection pump. The die temperature ranged from 175 to 190 °C. The cell structures of the samples at various die temperatures are listed in Figure S1 (from supplementary materials). In addition, the results obtained at 180 °C are shown in Figure 11. As expected, compared with pure TPEE, all the samples with PTFE nanofibrils show decreased cell diameter and higher cell density. Especially for samples PTFE3 and PTFE5, the cell diameter exhibits a dramatic decreasing trend together with a pronounced increase in cell density.

**Figure 12:**
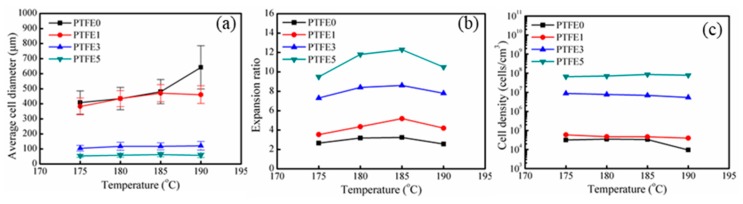
Statistical results of the foam morphologies: (**a**) average cell diameter, (**b**) expansion ratio, (**c**) cell density.

The statistical results of the foam morphology are shown in Figure 12. By introducing PTFE nanofibrils, the average cell diameter decreases and the cell density increases. When the die temperature was 185 °C, the average cell diameter drops from 480.7 μm for PTFE0 to 63.1 μm for PTFE5, whereas the cell density increases from 3.3 × 10^4^ to 8.6 × 10^7^ cells/cm^3^. Zhao’s work [38] also showed that by introducing PTFE fibrils, the cell sizes further decreased from 600 μm to 30 μm, with a dramatically increasing cell density by three orders of magnitude. The cell size distribution of all the foamed samples is shown in Figure S2 (from supplementary materials), the samples with PTFE nanofibrils show a much more uniform cell structure and narrower cell-size distribution. The increased cell density can be ascribed to the heterogeneous nucleation from the fibrils, which can act as cell nucleation sites and lower the free energy barrier of nucleation [27,29,31,33]. Moreover, as discussed for the rheological tests, the in-situ fibrillated PTFE can significantly change the rheological properties of the TPEE melts. In the extrusion foaming process, when the die temperature was 180 °C, the die pressure increased from 8.2 MPa for PTFE0 to 14.8 MPa for PTFE5 due to the highly entangled PTFE network. The higher die pressure leads to a higher pressure drop rate, which can dramatically improve the nucleation efficiency and increase the cell density [1].

After the samples are extruded from the die, the cells grow in the air and the temperature of the material decreases. The samples with PTFE nanofibrils show a higher crystallization temperature and a faster crystallization rate, which means that, during the cooling process, the TPEE crystals grow faster and uniformly. These newly generated crystals can also promote foaming. It is notable that the expansion ratio of the pure TPEE ranges from 2.5 to 3.5 fold and for PTFE5 it ranges from 9.5 to 12.4 fold. This pronounced improvement can be attributed to two factors. First, the cell walls experience a biaxial stretching process. When the cells grow in the pure TPEE, because of the absence of a branched structure or a fibril network, the elongational viscosity decreases during stretching and, therefore, the cells may break or collapse. In contrast, the viscosity of the TPEE with PTFE nanofibrils rises with strain. To some extent, the strain-hardening effect can prevent the cell walls from opening. Secondly, the fast growing TPEE crystals can make the cell walls stiff. Thus, much more CO_2_ can be trapped in the cells for cell growth, resulting in a higher expansion ratio. As a result of the improved crystallization process and better rheological properties, TPEE/PTFE nanocomposites show an evident enhancement of the foaming ability in extrusion processing. Further, nanofibrils also promote cell nucleation. Consequently, TPEE foams with higher cell density, smaller cell diameter, higher expansion ratio and more uniform cell structure are obtained with inclusion of PTFE nanofibrils.

## 4. Conclusions

TPEE/PTFE nanocomposites with in-situ fibrillated PTFE were successfully prepared using a twin-screw extruder. The long aspect ratio PTFE nanofibrils can significantly promote the crystallization of TPEE and refine the crystals. A high PTFE nanofibril content can form a network in the TPEE matrix and greatly change its rheological properties, especially for the elongational viscosity. The improvements in the crystallization and rheological properties enhanced the foaming ability of TPEE, which was tested by a continuous extrusion foaming process. With the inclusion of in-situ generated PTFE nanofibrils, the expansion ratio increased by near ten-fold when compared to the pure TPEE. The cells show a more uniform structure and a smaller average cell diameter.

## Figures and Tables

**Table 1:** Composition of thermoplastic polyether ester elastomer/ polytetrafluoroethylene (TPEE/PTFE) nanocomposites.

Sample	PTFE Content (wt %)	2,2′-BOZ Content (wt %)	*η*_0.01_^a^ (Pa·s)
PTFE0	0	0.5	752.4
PTFE1	1	0.5	993.5
PTFE3	3	0.5	1853.5
PTFE5	5	0.5	3467.6

*η*_0.01_^a^ The viscosities at 0.01rad/s of the products were obtained through small amplitude oscillation.

**Table 2:** Crystallization kinetic parameters for TPEE/PTFE nanocomposites.

Sample	Crystallization Onset Temperature (*T*_c, onset_ (°C))	Crystallization Temperature (*T*_c_ (°C))	Melting Temperature (*T*_m_ (°C))	Enthalpy of Crystallization (Δ*H*_c_ (J/g))	Crystallization Rate Constant (*k’*)	Avrami Exponent (*n*)	Half Crystallization Time (*t*_1/2_ (min))	Variance (*r*^2^)
PTFE0	180.4	164.3	197.1	28.33	0.80	1.52	4.78	0.9908
PTFE1	175.5	169.6	196.7	25.68	0.67	3.04	1.71	0.9990
PTFE3	177.1	170.6	196.8	25.12	0.68	2.87	1.73	0.9997
PTFE5	177.8	170.8	197.3	24.72	0.65	2.85	1.92	0.9996

## Supplementary Materials

The following are available online at https://www.mdpi.com/2073-4360/11/12/1983/s1, Figure S1: SEM images of the foamed TPEE/PTFE nanocomposites obtained with different die temperature. (a1: PTFE0, 175 °C; a2: PTFE0, 180 °C; a3: PTFE0, 185 °C; a4: PTFE0, 190 °C; b1: PTFE1, 175 °C; b2: PTFE1, 180 °C; b3: PTFE1, 185 °C; b4: PTFE1, 190 °C; c1: PTFE3, 175 °C; c2: PTFE3, 180 °C; c3: PTFE3, 185 °C; c4: PTFE3, 190 °C; d1: PTFE5, 175 °C; d2: PTFE5, 180 °C; d3: PTFE1, 185 °C; d4: PTFE5, 190 °C), Figure S2: Cell size distribution of the foamed TPEE/PTFE nanocomposites obtained with different die temperature. (a: 175 °C; b: 180 °C; c: 185 °C; d:190 °C).
