# Supplementary material for: Improving the Continuous Microcellular Extrusion Foaming Ability with Supercritical CO2 of Thermoplastic Polyether Ester Elastomer through In-Situ Fibrillation of Polytetrafluoroethylene
Source: Polymers (Basel). 2019 Dec 2;11(12):1983. doi: 10.3390/polym11121983 (PMC6960977; doi:10.3390/polym11121983)
Supplement: Supplementary file 1 [file polymers-11-01983-s001.pdf]

polymers

MDPI

Supplementary material:

Improving the Continuous Microcellular Extrusion Foaming Ability with Supercritical CO_2 of Thermoplastic Polyether Ester Elastomer through in-Situ Fibrillation of Polytetrafluoroethylene

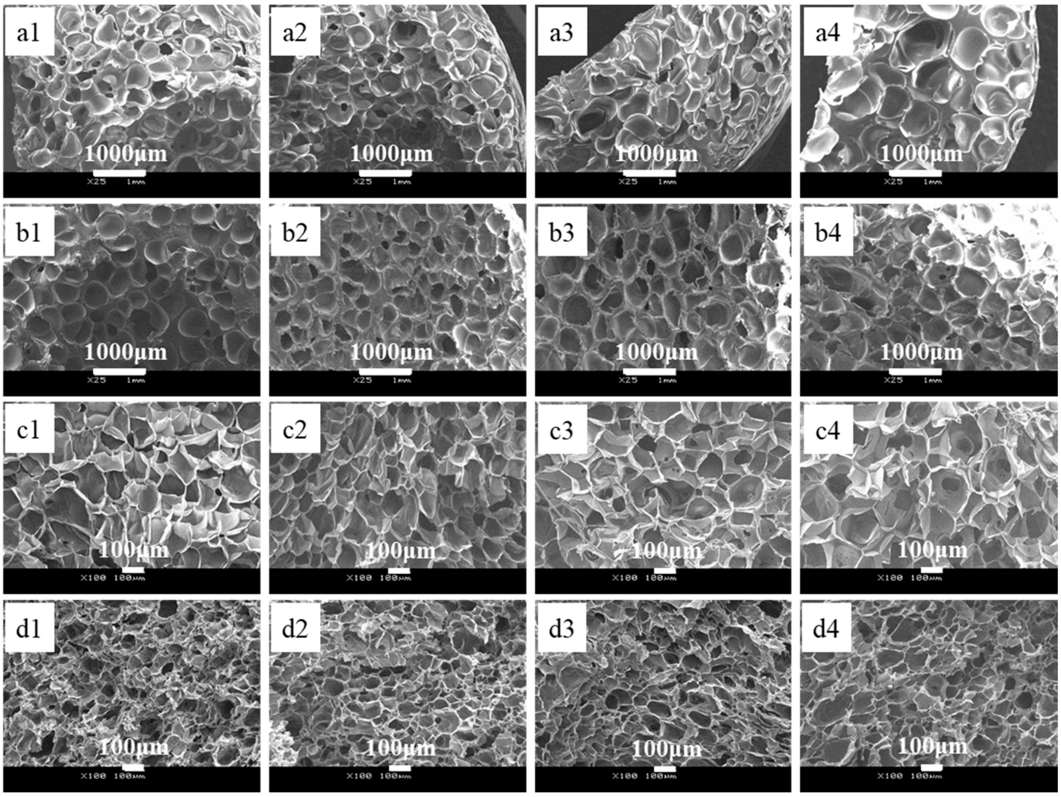

Figure S1. SEM images of the foamed TPEE/PTFE nanocomposites obtained with different die temperature. (**a1**: PTFE0, 175 °C; **a2**: PTFE0, 180 °C; **a3**: PTFE0, 185 °C; **a4**: PTFE0, 190 °C; **b1**: PTFE1, 175 °C; **b2**: PTFE1, 180 °C; **b3**: PTFE1, 185 °C; **b4**: PTFE1, 190 °C; **c1**: PTFE3, 175 °C; **c2**: PTFE3, 180 °C; **c3**: PTFE3, 185 °C; **c4**: PTFE3, 190 °C; **d1**: PTFE5, 175 °C; **d2**: PTFE5, 180 °C; **d3**: PTFE1, 185 °C; **d4**: PTFE5, 190 °C).

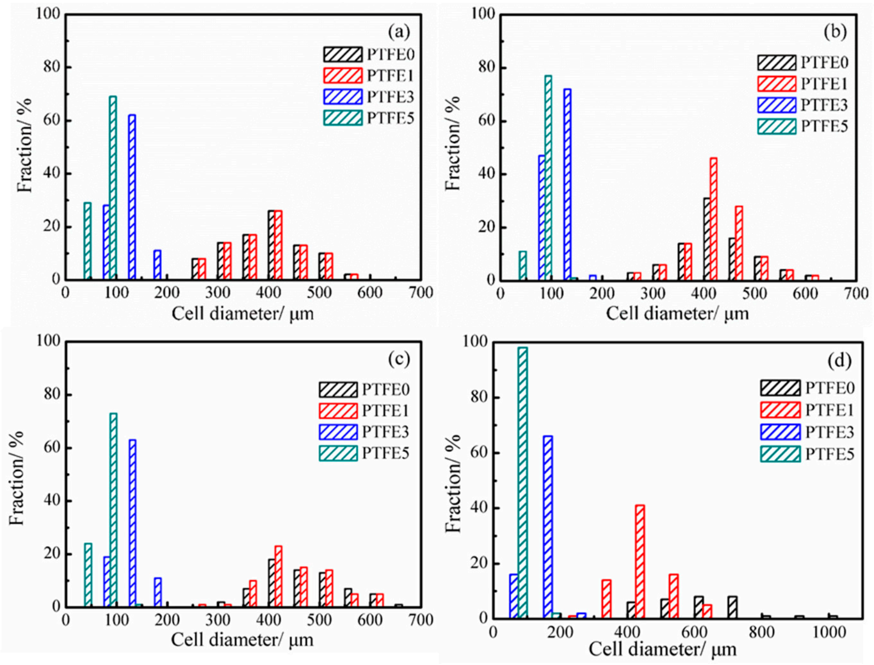

Figure S2. Cell size distribution of the foamed TPEE/PTFE nanocomposites obtained with different die temperature. (**a**: 175 °C; **b**: 180 °C; **c**: 185 °C; **d**:190 °C).
